# Association between the Fatty Liver Index (FLI) and incident coronary heart disease: insights from a cohort study on the Chinese population

**DOI:** 10.3389/fendo.2024.1367853

**Published:** 2024-12-20

**Authors:** Ying Miao, Yu Wang, Pijun Yan, Yi Li, Zhuang Chen, Nanwei Tong, Qin Wan

**Affiliations:** ^1^ Department of Endocrinology and Metabolism, Center for Diabetes and Metabolism Research, West China Hospital, Sichuan University, Chengdu, China; ^2^ Department of Endocrinology and Metabolism, Affiliated Hospital of Southwest Medical University, Luzhou, China; ^3^ Metabolic Vascular Disease Key Laboratory of Sichuan Province, Luzhou, China; ^4^ Sichuan Clinical Research Center for Diabetes and Metabolism, Luzhou, China; ^5^ Sichuan Clinical Research Center for Nephropathy, Luzhou, China; ^6^ Cardiovascular and Metabolic Diseases Key Laboratory of Luzhou, Luzhou, China; ^7^ Southwest Medical University, Luzhou, China; ^8^ Department of Cardiology, Luzhou People’s Hospital, Luzhou, China; ^9^ Experimental Medicine Center, the Affiliated Hospital of Southwest Medical University, Luzhou, China

**Keywords:** the Fatty Liver Index, coronary heart disease, metabolic dysfunction-associated steatotic liver disease, REACTION project, Chinese population

## Abstract

**Background:**

The debate persists regarding whether metabolic dysfunction-associated steatotic liver disease (MASLD) actively contributes to coronary heart disease or merely acts as a passive indicator.

**Objective:**

This research aims to clarify the relationship between liver fat accumulation, as quantified by FLI, and the risk of developing coronary heart disease.

**Methods:**

Conducted from April to November 2011, the REACTION project, spearheaded by the Endocrinology Branch of the Chinese Medical Association, focused on Chinese adults aged 40 and above. Comprehensive data collection employed both questionnaires and specialized medical equipment, covering physical measurements, blood pressure, and pertinent biochemical markers. The study population excluded those with pre-existing coronary heart disease and acute myocardial infarction. Based on the initial data, participants were segmented and grouped into three categories. Analytically, the study utilized Cox proportional hazards models, further enhanced by stratified analyses to identify variations within predefined demographic groups.

**Results:**

In this study, we enrolled 8,647 participants, comprising 2,887 males and 5,760 females. Over the 10-year non-interventional follow-up period, 433 participants (5%) passed away due to various reasons, with 55 deaths attributed to coronary heart disease/myocardial infarction, accounting for 12.7% of total deaths. Additionally, 484 participants were diagnosed with new-onset coronary heart disease, resulting in an incidence rate of 5.5%. Spearman correlation analysis revealed a positive correlation between FLI and traditional risk factors for coronary heart disease, including age, male gender, abnormal glucose metabolism, hypertension, smoking, TG, TC, LDL-C, etc. The Log-rank test indicated a rising cumulative incidence of coronary heart disease with increasing FLI groupings (P<0.01). Moreover, Cox regression analysis highlighted a notable correlation between FLI levels as a risk factor and the onset of coronary heart disease. After adjusting for other risk factors, individuals in the 30≤FLI<60 group exhibited a 1.203-fold higher risk of coronary heart disease compared to those in the FLI<30 group (p=0.126), while participants in the FLI≥60 group had a 1.386-fold higher risk than those in the FLI<30 group (p=0.041).

**Conclusion:**

Elevated FLI values are strongly associated with an increased susceptibility to coronary heart disease, indicating its potential value as a prognostic marker for the condition.

## Introduction

In recent years, the prevalence of metabolic dysfunction-associated steatotic liver disease (MASLD) has escalated, affecting an estimated 30% of the global population (1). MASLD is defined as hepatic steatosis and one or more of the five cardiometabolic risk factors: i) body mass index (BMI) ≥25 kg/m2 (≥23 kg/m 2 for Asians) or waist circumference >94 cm for males and >80 cm for females or ethnicity adjusted; ii) fasting serum glucose ≥5.6 mmol/L (100 mg/dL) or 2-hour post-load glucose levels ≥7.8 mmol/L (≥140 mg/dL) or glycated haemoglobin ≥5.7% (39 mmol/L) or type 2 diabetes or treatment for type 2 diabetes; iii) blood pressure ≥130/85 mmHg or specific antihypertensive drug treatment; iv) plasma triglycerides ≥1.70 mmol/L (150 mg/dL) or lipid-lowering treatment; and v) plasma high-density lipoprotein (HDL) cholesterol ≤1.0 mmol/L (40 mg/dL) for males and ≤1.3 mmol/L (50 mg/dL) for females or lipid-lowering treatment. Patients with steatotic liver disease and at least one of the cardiometabolic risk factors are categorized as MASLD when they have no other causes of steatosis (2). Formerly known as non-alcoholic fatty liver disease, MASLD is characterized by liver fat accumulation exceeding 5% in the absence of excessive alcohol consumption, chronic liver diseases, or other factors causing liver damage (3). This condition often serves as the primary contributor to hepatic functional abnormalities, subsequently leading to liver fibrosis, cirrhosis, and hepatocellular carcinoma. A growing body of research indicates that MASLD is not solely a liver-centric condition but also a multisystemic disorder, elevating the risk for liver-related diseases while also being associated with the development of other systemic conditions. Notably, cardiovascular disease emerges as the leading cause of mortality among MASLD patients (4).

Coronary heart disease stands as a predominant cardiovascular affliction on a global scale. Current statistics reveal an alarming annual mortality rate of approximately 7.4 million attributed to this condition (5), imposing significant economic burdens on societies worldwide. While epidemiological evidence consistently underscores the link between MASLD and cardiovascular diseases, the specific role MASLD plays in the progression of cardiovascular disease warrants further investigation and discourse (4).

In large-scale cohort studies, the Fatty Liver Index (FLI) has emerged as a reliable tool for identifying liver fat accumulation in study participants. The widespread adoption of this index can be attributed to its non-invasive methodology and high degree of accuracy. Consequently, the present research employs cohort analysis to explore the relationship between FLI levels and the risk of coronary heart disease onset, aiming to furnish novel epidemiological evidence regarding the impact of early-stage hepatic fat deposition on the elevated risk of coronary heart disease.

## Methods

### Study population

The study population was derived from the REACTION study, predominantly comprising participants from Luzhou City in Sichuan Province. From April to November 2011, we recruited 10,150 individuals, all aged 40 or older. Eligibility criteria stipulated that participants must have lived in Luzhou for a minimum of five years, shown a commitment to follow-up procedures, and be at least 40 years old. Exclusion criteria encompassed past instances of coronary heart disease, a history of myocardial infarction, age under 40, prolonged heavy alcohol consumption, mobility impairments, unwillingness to engage in follow-up, and data incompleteness. After meticulous screening, 8,647 individuals met the set criteria and became part of our study cohort.

In 2021, when conducting follow-up evaluations centered on the endpoint of coronary heart disease, we primarily depended on chronic disease data provided by both the Health Commission and the Disease Control and Prevention Center of Luzhou City.

### Data collection and assessment

Trained research personnel conducted face-to-face interviews and administered standardized questionnaires to collect demographic information on participants, such as age and gender, as well as data on their lifestyle behaviors, including smoking habits and dietary patterns, medical histories, and medication usage. Prior to breakfast, measurements of the participants’ weight, height, waist circumference, and hip circumference were taken, and the Body Mass Index (BMI) was subsequently calculated. Seated systolic blood pressure (SBP) and diastolic blood pressure (DBP) were measured every 5 minutes across all participants, with each individual undergoing three readings; the average of these measurements was then determined. Participants fasted for a minimum of 10 hours before undergoing a 75g oral glucose tolerance test. Blood samples were collected at 0 and 2 hours post-test and stored at -80°C. Blood sugar measurements included glycated hemoglobin A1c (HbA1c), fasting blood glucose (FBG), and 2-hour postprandial blood glucose (PBG). Lipid profile assessments covered low-density lipoprotein cholesterol (LDL-C), high-density lipoprotein cholesterol (HDL-C), total cholesterol (TC), and triglycerides (TG). All laboratory analyses were conducted in a certified central laboratory in compliance with the International Organization for Standardization (ISO) 15189 guidelines.

### Definitions

In accordance with prior studies (6), the Fatty Liver Index (FLI) was determined using the equation:FLI = (e^[0.953 × ln(TG) + 0.139 × BMI + 0.718 × ln(GGT) + 0.053 × WC - 15.745]^)/(1 + e^[0.953 × ln(TG) + 0.139 × BMI + 0.718 × ln(GGT) + 0.053 × WC - 15.745]^) × 100. Here, triglyceride levels are denoted in mg/dl, gamma-glutamyl transferase(GGT) is measured in U/l, and WC is recorded in cm. Hypertension is defined by the WHO as having SBP ≥ 140 mmHg, DBP ≥ 90 mmHg, or being on antihypertensive medication (7). Diagnostic standards for normal glucose metabolism(NGT), prediabetes, and diabetes(DM) adhere to previously established criteria (8). Coronary heart disease diagnosis is predicated on findings from coronary angiography, with a narrowing exceeding 50% in the luminal diameter of at least one major coronary artery indicating a positive result (9).

### Statistical analysis

The sample’s primary characteristics were presented using descriptive statistics. Continuous variables, based on their distribution, are detailed as mean ± standard deviation (SD) or median (interquartile range, IQR). Categorical variables are depicted as counts (percentages). Continuous variable comparisons utilized the Student’s t-test, Mann-Whitney U test, Kruskal-Wallis H test, or one-way ANOVA, contingent on data normality. Chi-square tests were employed for inter-group categorical variable comparisons. In all statistical evaluations, p-values were two-tailed, with significance set at p < 0.05. SPSS software (version 26.0) facilitated all analyses.

Subjects were categorized into three FLI groups at baseline: FLI < 30 indicated no MASLD; 30 ≤ FLI < 60 represented intermediate FLI; and FLI ≥ 60 denoted MASLD. Baseline characteristics across these groups were compared. Spearman correlation coefficients were performed to assess whether there was an association between FLI and other variables. The cumulative coronary heart disease incidence was determined by dividing cases by the total subjects in each FLI category. Cox proportional hazards regression models assessed the coronary heart disease risk associated with each baseline FLI level.

## Results

### Baseline characteristics

In this study, we conducted a baseline evaluation of 8,647 participants, including 2,887 males and 5,760 females. Over the course of a 10-year non-interventional follow-up, 433 participants (5%) passed away due to various causes. Among them, 55 individuals died from coronary heart disease/myocardial infarction, accounting for 12.7% of the deceased; 59 of the deceased were diagnosed with coronary heart disease, representing 13.6% of the deceased. Furthermore, 484 participants developed new-onset coronary heart disease, leading to an incidence rate of 5.5%. **Table 1** presents the baseline demographic and clinical characteristics of these participants. When categorized by FLI, significant differences were observed in terms of gender, age, glucose metabolism, BMI, waist circumference (WC), hip circumference, lipid profiles, liver enzyme levels, serum creatinine (Cr) levels, pulse, smoking habits, drinking tea, drinking, hypertension prevalence, eating eggs, dairy products, soy products, consuming viscera, eating sweets, consuming salt, and educational level.

**Table 1:** Baseline Characteristics of the Subjects Based on FLI Categories.

groups Variables	FLI<30 (n=5497)	30≤FLI<60 (n=2116)	FLI≥60 (n=1034)	Test the statistical value	p-value
Male (%)	1606(29.2%)	808(38.2%)	437(45.7%)	135.906	<0.001
Age(years)	57.00(50.00,65.00)	60.00(54.00,66.00)	59.00(52.00,66.00)	82.773	<0.001
Glycemic status (normal/prediabetes/diabetes)	3490/1377/630	895/695/526	349/319/366	624.912	<0.001
BMI(kg/m2)	22.30(20.70,24.00)	25.70(24.20,27.30)	27.90(26.10,29.90)	3429.936	<0.001
WC(cm)	78.40(73.10,84.00)	89.00(84.23,93.00)	95.45(90.08,100.00)	3272.092	<0.001
Hip circumference(cm)	91.20(87.00,95.40)	98.00(94.00,102.00)	102.00(97.38,107.00)	2372.720	<0.001
TG(mmol/l)	1.04(0.80,1.39)	1.71(1.28,2.27)	2.34(1.69,3.28)	2750.602	<0.001
TC(mmol/l)	4.37(3.63,5.14)	4.79(4.09,5.52)	4.89(4.18,5.62)	323.495	<0.001
HDL-C(mmol/l)	1.28(1.04,1.52)	1.17(0.97,1.36)	1.10(0.93,1.27)	346.755	<0.001
LDL-C(mmol/l)	2.42(1.91,2.98)	2.71(2.18,3.29)	2.67(2.11,3.23)	210.799	<0.001
ALT(U/L)	11.00(8.00,15.00)	15.00(11.00,22.00)	20.00(14.00,28.00)	1344.175	<0.001
AST(U/L)	18.00(15.00,22.00)	21.00(17.00,26.00)	24.00(19.00,31.00)	713.024	<0.001
GGT(U/L)	14.00(11.00,20.00)	25.00(18.00,39.00)	41.00(27.00,73.00)	2708.699	<0.001
Cr(μmol/L)	60.60(53.90,68.10)	64.50(57.50,72.70)	66.90(59.10,76.40)	337.522	<0.001
Pulse(beats per minute)	77.00(71.00,85.00)	78.00(72.00,86.00)	80.00(73.00,87.00)	30.409	<0.001
Smokingn(%)	702(12.7%)	337(17%)	194(19.8%)	28.931	<0.001
Current drinking tea(%)	2748(50.90%)	1141(55.20%)	617(61.00%)	40.154	<0.001
Current drinking(%)	1383(26.90%)	630(31.50%)	366(37.20%)	49.130	<0.001
Hypertension(%)	753(13.6%)	383(18.1%)	217(21%)	47.856	<0.001
Family history of diabetes(%)	537(9.80%)	190(9.00%)	108(10.40%)	1.929	0.381
Eat fresh fruit(%)	182(3.30%)	89(4.20%)	33(3.20%)	5.044	0.283
Consumption of aquatic products(%)	4907(89.30%)	1889(89.30%)	923(89.30%)	5.703	0.222
Eat eggs(%)	4676(85.10%)	1763(83.30%)	858(83.00%)	9.959	0.041
Eat dairy products(%)	3377(61.40%)	1293(61.10%)	582(56.30%)	13.955	0.007
Eat soy products(%)	4712(85.70%)	1775(83.90%)	861(83.30%)	9.577	0.048
Eat fried food(%)	1598(29.10%)	602(28.40%)	305(29.50%)	4.809	0.307
Feeder viscera(%)	2061(37.50%)	710(33.60%)	354(34.20%)	15,511	0.004
Eat sweets(%)	3307(60.20%)	1172(55.40%)	571(55.20%)	21.259	<0.001
Take salt(%)	3921(71.30%)	1533(72.40%)	764(73.90%)	11.124	0.025
Physical activity(%)	4030(73.30%)	1598(75.50%)	748(72.30%)	5.144	0.273
Educational level(小/初/高)	1808/1975/1651	776/735/576	383/376/264	19.056	0.004

BMI, body mass index; WC, waist circumference; TG, triglycerides; TC, total cholesterol; HDL-C, high-density lipoprotein cholesterol; LDL-C, low-density lipoprotein cholesterol; ALT, alanine aminotransferase; AST, aspartate aminotransferase; GGT, gamma-glutamyl transferase; Cr, creatinine.

### Association between FLI and clinical/laboratory characteristics

**Table 2** presents the association of FLI with clinical and laboratory characteristics in all participants, analyzed using Spearman correlation coefficients. The results indicated that FLI was positively associated with age, male gender, glycemic status, hypertension, current smoking, current tea drinking, current alcohol consumption, salt intake, pulse, weight, WC, hip circumference, triglycerides (TG), total cholesterol (TC), low-density lipoprotein cholesterol (LDL-C), alanine aminotransferase (ALT), aspartate aminotransferase (AST), gamma-glutamyl transferase (GGT), and Cr, while it was negatively associated with fresh fruit consumption, egg consumption, dairy product consumption, fried food consumption, viscera consumption, sweet consumption, and high-density lipoprotein cholesterol (HDL-C) levels (P<0.05).

**Table 2:** Association between FLI and Clinical/Laboratory Characteristics.

	r	P value
Age	0.146	<0.001
Gender(female vs male)	-0.146	<0.001
Glycemic status	0.237	<0.001
Hypertension(no vs yes)	-0.090	<0.001
Family history of diabetes(no vs yes)	-0.001	0.952
Current smoking(no vs yes)	-0.073	<0.001
Current drinking tea(no vs yes)	-0.067	<0.001
Current drinking(no vs yes)	-0.078	<0.001
Educational level	-0.059	<0.001
Eat fresh fruit(no vs yes)	0.003	0.758
Consumption of aquatic products(no vs yes)	-0.015	0.165
Eat eggs(no vs yes)	0.025	0.018
Eat dairy products(no vs yes)	0.022	0.038
Eat soy products(no vs yes)	0.015	0.168
Eat fried food(no vs yes)	0.002	0.884
Feeder viscera(no vs yes)	0.041	<0.001
Eat sweets(no vs yes)	0.045	<0.001
Take salt(no vs yes)	-0.021	0.048
Physical activity(no vs yes)	-0.012	0.268
Pulse	0.055	<0.001
Weight	0.682	<0.001
WC	0.795	<0.001
Hip circumference	0.633	<0.001
TG	0.695	<0.001
TC	0.264	<0.001
HDL-C	-0.212	<0.001
LDL-C	0.239	<0.001
ALT	0.459	<0.001
AST	0.326	<0.001
GGT	0.645	<0.001
Cr	0.259	<0.001

### Log-rank test

**Figure 1:**
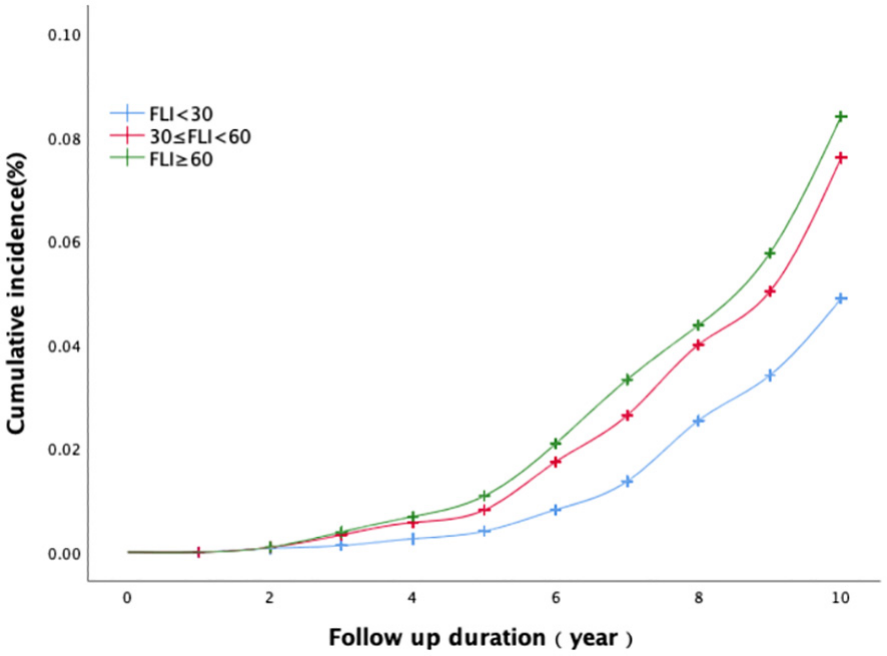
The association between baseline FLI categories and the cumulative incidence of coronary heart disease.

The Log-rank test (illustrated in **Figure 1**) demonstrates the temporal evolution of the cumulative incidence rate of coronary heart disease. Based on the Log-rank test results, an elevated FLI group corresponded to a higher cumulative incidence rate of coronary heart disease (χ2 = 28.701, P<0.01). Furthermore, the disparity in cumulative incidence rates between the three groups grew progressively over time.

### Analysis of coronary heart disease determinants

**Table 3** displayed the associations of FLI and other variables with the risk of presence of coronary heart disease. The univariate revealed that Age, Gender, Glycemic status, Hypertension, drinking, Consumption of aquatic products, Feeder viscera, Eat sweets, Physical activity, Pulse, Weight, WC, Hip circumference, TG, HDL-C, GGT, Cr and FLI were significantly associated with the development of coronary heart disease. Multivariable regression analysis showed that age, Hypertension, Pulse, HDL-C, and FLI were significantly associated with the development of coronary heart disease among the middle-aged and elderly population. Notably, each SD increase in FLI was associated with a significant 1.007-fold increased odds of coronary heart disease (95% CI, 1.002–1.012, P<0.05).

**Table 3:** Univariate and multivariate analysis of determinants of coronary heart disease in study subjects.

Variable	Univariate analysis		Multivariate analysis	
	Test the statistical value	P	HR(95%CI)	P
Age	-13.801	<0.001	1.059(1.049,1.070)	<0.001
Gender(male vs female)	12.336	<0.001	1.271(0.999,1.616)	0.051
Glycemic status	112.136	<0.001	0.991(0.949,1.034)	0.665
Hypertension(yes vs no)	25.556	<0.001	1.353(1.088,1.638)	0.007
Family history of diabetes	0.456	0.500		
Current smoking(yes vs no)	2.094	0.148		
Current drinking tea(yes vs no)	0.423	0.515		
Current drinking(yes vs no)	5.249	0.022	1.264(0.998,1.601)	0.052
Educational level	4.039	0.257		
Eat fresh fruit(yes vs no)	3.304	0.192		
Consumption of aquatic products(yes vs no)	13.825	0.001	1.157(0.872,1.535)	0.314
Eat eggs(yes vs no)	0.411	0.814		
Eat dairy products(yes vs no)	4.346	0.114		
Eat soy products(yes vs no)	0.687	0.709		
Eat fried food(yes vs no)	3.804	0.149		
Feeder viscera(yes vs no)	18.681	<0.001	1.065(0.876,1.294)	0.529
Eat sweets(yes vs no)	9.049	0.011	1.073(0.891,1.293)	0.457
Take salt(yes vs no)	3.511	0.173		
Physical activity(yes vs no)	10.505	0.005	0.899(0.734,1.101)	0.303
Pulse	-2.009	0.045	1.006(1.000,1.012)	0.048
Height	-0.344	0.731		
Weight	-4.035	<0.001	0.998(0.984,1.013)	0.805
WC	-7.8	<0.001		
Hip circumference	-4.715	<0.001	1.008(0.993,1.023)	0.283
TG	-2.703	0.007		
TC	-0.314	0.753		
HDL-C	-5.084	<0.001	0.714(0.527,0.969)	0.031
LDL-C	-0.857	0.391		
ALT	-0.708	0.479		
AST	-0.568	0.57		
GGT	-3.322	0.001		
Cr	-4.402	<0.001	0.999(0.995,1.004)	0.764
FLI	-6.32	<0.001	1.007(1.002,1.012)	0.008

### Cox proportional hazards regression analysis

**Table 4** presents the outcomes of the Cox proportional hazards regression analysis on the association between FLI levels and the onset of coronary heart disease. Based on their initial FLI measurements, participants were divided into three distinct categories: FLI<30, 30≤FLI<60, and FLI≥60. Model 1 stands as the foundational model, unadjusted for any additional variables. When juxtaposed with the FLI<30 category, the coronary heart disease risk escalated by 1.553 times in the 30≤FLI<60 group(P<0.001) and 1.719 times in the FLI≥60 group(P<0.001). Model 2 factored in gender and age. Notably, the likelihood of coronary heart disease within the 30≤FLI<60 and FLI≥60 groups was elevated by 1.406(P=0.001) and 1.639(P<0.001) times compared to the FLI<30 category, respectively. For Model 3, adjustments were incorporated for gender and age, alongside other variables such as glycemic status, drinking, Consumption of aquatic products, Feeder viscera, Eat sweets, Physical activity, Pulse, Weight, Hip circumference, HDL-C, Cr and a history of hypertension. When contrasted with the FLI<30 cohort, the probabilities of coronary heart disease in the 30≤FLI<60 and FLI≥60 brackets increased by 1.203(P=0.126) and 1.386(P=0.041) times, respectively.

**Table 4:** Cox proportional hazard regression model for FLI and the risk of new onset coronary heart disease.

groups	Model1			Model2			Model3		
	HR	95%CI	P	HR	95%CI	P	HR	95%CI	P
FLI<30	1			1			1		
30≤FLI<60	1.553	(1.269∼1.900)	<0.001	1.406	(1.149∼1.721)	0.001	1.203	(0.949∼1.524)	0.126
FLI≥60	1.719	(1.337∼2.210)	<0.001	1.639	(1.274∼2.109)	<0.001	1.386	(1.014,1.896)	0.041

Model1 is not adjusted for any variables; Model2 is adjusted for gender and age; Model3 is further adjusted for glycemic status, drinking, Consumption of aquatic products, Feeder viscera, Eat sweets, Physical activity, Pulse, Weight, Hip circumference, HDL-C, Cr and a history of hypertension.

### Results of stratified analysis

**Figure 2:**
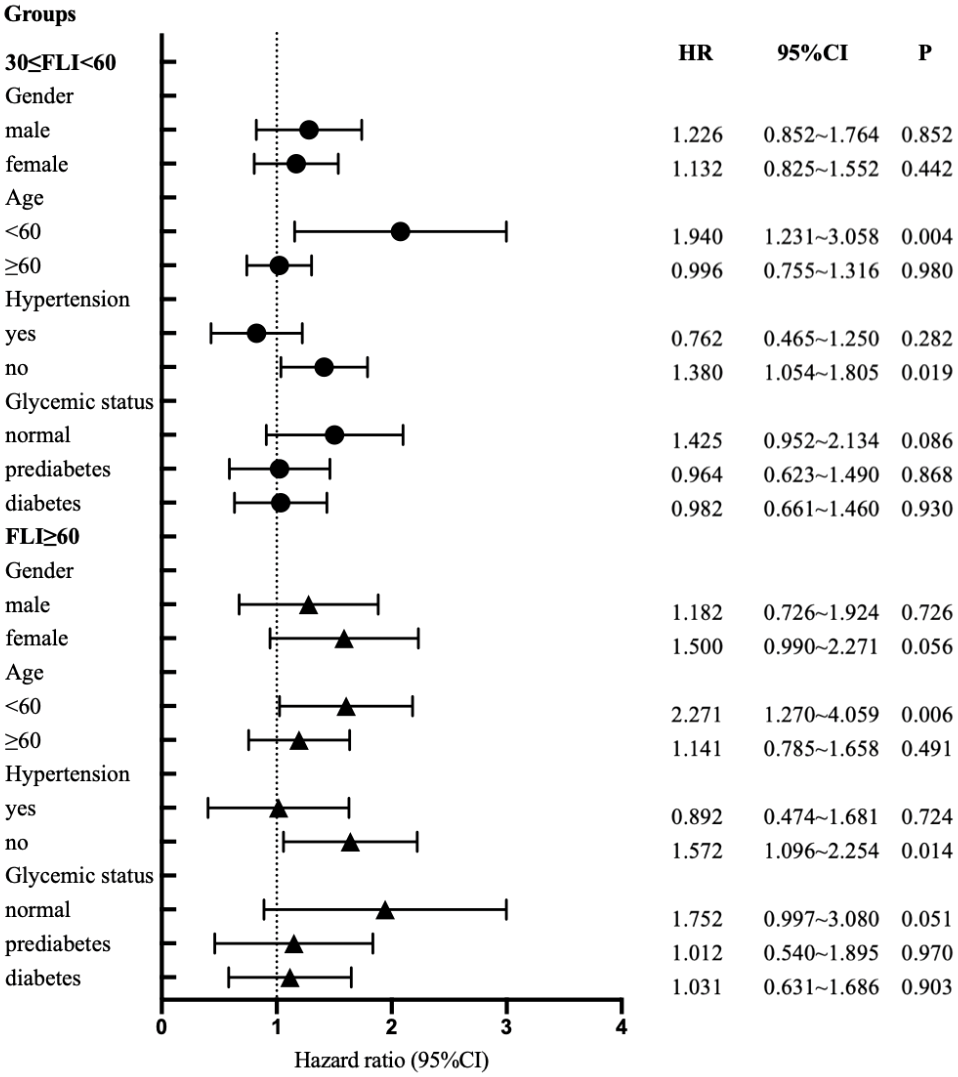
Cox proportional - hazards regression models for regression predefined stratification subpopulations.

**Figure 2** presents a stratified analysis, categorizing participants by gender, age, hypertension, and glucose metabolism. The diagram elucidates how baseline FLI correlates with the risk of developing coronary heart disease across different subgroups. Upon adjusting for various factors, the findings indicate an elevated risk of coronary heart disease in both the 30≤FLI<60 and FLI≥60 groups when compared to the FLI<30 group. Notably, this increase was statistically significant in subgroups aged under 60 and those without hypertension (p<0.05).

## Discussion

In this study, we examined 8,647 adults aged 40 and above from the Luzhou region. Our findings indicate that FLI is positively correlated with traditional risk factors for coronary heart disease such as age, male gender, abnormal glucose metabolism, hypertension, smoking, TG, TC, LDL-C, etc. Moreover, individuals with elevated FLI face an increased risk of coronary heart disease, with the high FLI group showing a significantly higher risk than the low FLI group. Upon stratified analysis, after accounting for gender, age, and hypertension status, individuals with an FLI≥30 exhibit an increased risk of coronary heart disease. This association between abnormal FLI and coronary heart disease risk is particularly pronounced among individuals younger than 60 years and those without hypertension. Specifically, among the non-hypertensive population and those under 60, the link between abnormal FLI and the risk of coronary heart disease is notably significant.

In previous studies, an FLI<30 was considered normal, while an FLI≥60 was identified as a marker for fatty liver disease (6). This suggests that when the FLI is at or above 30, potential abnormalities may be present. In research conducted by Chung GE (10) and his team, they defined fatty liver disease using an FLI≥60. Their findings revealed that individuals with an FLI≥60 had a higher risk of mortality from cardiovascular diseases and overall mortality. This implies that deviations in FLI levels are associated with increased mortality risks across a range of diseases.

Our study’s results are in close agreement with the findings presented by Lee HH et al (11). Their study involved a large cohort of 9,775,066 adults aged 20 to 79 years. They found that individuals with an FLI≥30 had a significantly increased risk of cardiovascular diseases, including conditions such as myocardial infarction, ischemic stroke, heart failure, and cardiovascular-related mortality, compared to those with an FLI<30. In contrast, while Lee HH et al.’s research mainly focused on the general relationship between FLI and cardiovascular diseases, our study concentrated specifically on the connection between FLI and coronary heart disease. Although an FLI≥30 is not currently a definitive diagnostic criterion for fatty liver disease, its association with cardiovascular diseases is evident. In our study, after multifactorial regression analysis, certain trends were observed in some stratified subsets. However, these differences did not reach statistical significance, likely due to the smaller sample size in specific subgroups after stratification. Nevertheless, it’s crucial to highlight that in the population over 40 years old, the risk of coronary heart disease rises with increasing FLI levels.

Our findings indicate a more significant correlation between FLI and coronary heart disease in individuals under 60 years old and those without hypertension. We attribute this to the large inter-individual heterogeneity in coronary heart disease risk, which is only partially explained by traditional risk factors used in primary risk assessment. Residual, unobserved heterogeneity leads to the attenuation of hazard rates with age and an underestimation of hazard ratios (12). Therefore, in individuals over 60 years old, unobserved heterogeneity may diminish the correlation between FLI and coronary heart disease. Additionally, we hypothesize that this heterogeneity may also be relevant in the hypertensive population. Hypertensive individuals often exhibit various metabolic abnormalities, influenced by multiple genetic and environmental factors, which collectively contribute to an increase in coronary heart disease risk factors, thereby weakening the correlation between FLI and coronary heart disease.

Regarding the mechanism of MASLD involvement in coronary heart disease, existing research indicates a correlation between MASLD and risk factors such as diabetes (13) and hypertension (14). Furthermore, numerous studies have shown that MASLD is associated with insulin resistance and metabolic disorders, thereby increasing the risk of coronary heart disease (15–17). Moreover, research has demonstrated that the pathogenesis of coronary heart disease involving MASLD, particularly the activation of the renin-angiotensin system (18), correlates with factors such as lipid metabolism, insulin resistance, and reactive oxygen species production, thereby accelerating the progression of coronary heart disease (19–23).

Our research benefits from a 10-year non-interventional cohort design; however, it also possesses inherent limitations. Initially, we excluded individuals diagnosed with coronary heart disease at the outset, which may have introduced selection bias. Furthermore, the endpoint event data are predominantly sourced from registrations by health departments and disease control centers, prompting concerns regarding potential underreporting.

As previously mentioned, it’s crucial to highlight that the risk of coronary heart disease significantly increases when FLI is ≥30 and further elevates with rising FLI values. Therefore, individuals with an FLI≥30 require particular attention, and proactive interventions are imperative to reduce their risk of coronary heart disease.

## Conclusion

Elevated FLI values are strongly associated with an increased susceptibility to coronary heart disease, indicating its potential value as a prognostic marker for the condition.

## Data availability statement

The raw data supporting the conclusions of this article will be made available by the authors, without undue reservation.

## Ethics statement

The present study was approved by the Research Ethics Committees of the Rui-Jin Hospital affiliated to the Jiao-Tong University School of Medicine and also by the affiliated Hospital of Southwest Medical University. Patient/participant [legal guardian/next of kin] provided written informed consent to participate in this study.

## Author contributions

YM: Writing – review & editing, Writing – original draft, Methodology, Investigation, Conceptualization. YW: Writing – original draft, Methodology, Investigation, Conceptualization. PY: Writing – original draft, Methodology, Investigation. YL: Writing – original draft, Methodology, Investigation. ZC: Writing – original draft, Methodology. NT: Writing – review & editing, Conceptualization. QW: Writing – review & editing, Supervision, Funding acquisition.
